# Long-Term Prognostic Impact of Stress Hyperglycemia in Non-Diabetic Patients Treated with Successful Primary Percutaneous Coronary Intervention

**DOI:** 10.3390/jpm14060591

**Published:** 2024-05-31

**Authors:** Lidija Savic, Igor Mrdovic, Milika Asanin, Sanja Stankovic, Ratko Lasica, Gordana Krljanac, Damjan Simic, Dragan Matic

**Affiliations:** 1 Faculty of Medicine, University of Belgrade, 11000 Belgrade, Serbia; igormrd@gmail.com (I.M.); masanin2013@gmail.com (M.A.); drlasica@gmail.com (R.L.); gkrljanac@gmail.com (G.K.); dragan4m@gmail.com (D.M.); 2 Emergency Center, Cardiology Intensive Care Unit & Cardiology Clinic, University Clinical Center of Serbia, 11000 Belgrade, Serbia; simicdamjan@hotmail.com; 3 Emergency Center, Center for Medical Biochemistry, University Clinical Center of Serbia, 11000 Belgrade, Serbia; sstankovic2013@gmail.com

**Keywords:** stress hyperglycemia, myocardial infarction, prognosis

## Abstract

Background: stress hyperglicemia (SH) is common in patients with ST-elevation myocardial infraction (STEMI). The aims of this study were to analyze the impact of SH on the incidence of all-cause mortality and major adverse cardiovascular events (MACE-cardiovascular death, nonfatal reinfarction, target vessel revascularization, and stroke) in STEMI patients without diabetes mellitus (DM) who have been treated successfully with primary PCI (pPCI). Method: we analyzed 2362 STEMI patients treated with successful pPCI (post-procedural flow TIMI = 3) and without DM and cardiogenic shock at admission. Stress hyperglycemia was defined as plasma glucose level above 7.8 mmol/L at admission. The follow-up period was 8 years. Results: incidence of SH was 26.9%. Eight-year all-cause mortality and MACE rates were significantly higher in patients with SH, as compared to patients without SH (9.7% vs. 4.2%, *p* < 0.001, and 15.7% vs. 9.4%, *p* < 0.001). SH was an independent predictor of short- and long-term all-cause mortality (HR 2.19, 95%CI 1.16–4.18, and HR 1.99, 95%CI 1.03–3.85) and MACE (HR 1.49, 95%CI 1.03–2.03, and HR 1.35, 95%CI 1.03–1.89). Conclusion: despite successful revascularization, SH at admission was an independent predictor of short-term and long-term (up to eight years) all-cause mortality and MACE, but its negative prognostic impact was stronger in short-term follow-up.

## 1. Introduction

Stress hyperglycemia (SH) is defined as a transitory increase in the blood glucose level that occurs in critically ill patients in intensive care units [1,2,3,4]. As a stress state, acute myocardial infarction (AMI) can lead to stress hyperglycemia [2,3]. It has been reported that around 20–50% of the patients with AMI and without diabetes mellitus (DM) exhibit acute hyperglycemia in response to physiological stress [5,6,7]. Previous studies have shown that hyperglycemia is associated with a poorer short-term prognosis in patients with AMI irrespective of their diabetic status [1,2,6,7,8,9,10,11,12,13,14,15,16]. There are data confirming that acute (stress) hyperglycemia in patients without DM has a worse prognostic impact after AMI (mortality and/or other adverse events), as compared to the prognostic impact of DM itself, which indicates that acute fluctuations in blood sugar levels are prognostically less favorable, as compared to chronically elevated values [16]. SH is also a predictor of the slow-flow/no-reflow phenomenon in patients undergoing percutaneous coronary intervention (PCI) [8,17], as well as a risk factor for stent thrombosis in these patients [6,7,11].

Many studies published so far have dealt with the prognostic impact of hyperglycemia in patients with AMI, i.e., patients with acute coronary syndrome (ACS) with different access to revascularization therapy [8]. The prognostic significance of SH in both diabetic and nondiabetic patients was analyzed [13], but the definition of SH in diabetic patients and the relationship between diabetes mellitus and SH remain controversial.

Primary percutaneous coronary intervention (pPCI) is the recommended and most effective therapeutic option in patients with STEMI. Studies have shown that successful angiographic reperfusion, which is defined as TIMI grade 3 flow through the infarct-related artery (IRA), is associated with good short- and long-term outcomes [18]. However, despite timely and successful reperfusion, some STEMI patients still have a high risk of adverse events because a certain amount of myocardial necrosis is inevitable. Therefore, it is necessary to define additional prognostic parameters in patients with STEMI who have undergone successful pPCI. To the best of our knowledge, the prognostic significance of SH in nondiabetic STEMI patients treated successfully with pPCI has not been analyzed so far.

The present study aims to analyze the impact of SH at admission on the incidence of all-cause mortality and major adverse cardiovascular events (MACE) in short- and long-term follow-up (up to eight years) in STEMI patients without DM who have been treated successfully with primary PCI.

## 2. Materials and Methods

### 2.1. Study Population, Inclusion and Exclusion Criteria, Data Collection, and Definitions

The present study enrolled 2362 consecutive patients hospitalized between December 2005 and January 2012, registered in the prospective University Clinical Center of Serbia STEMI Register. The purpose of the prospective University Clinical Center of Serbia STEMI Register has been published elsewhere [19]. The objective of the Register is to gather data on the management and short- and long-term outcomes of patients with STEMI treated with primary PCI. All consecutive STEMI patients aged 18 or older who were admitted to the Coronary Care Unit after being treated with pPCI in the Catheterization Lab of the Center were included in the Register. All the patients included received written information about their participation in the Register and the long-term follow-up, and their verbal and written consent was obtained [19].

**Figure 1:**
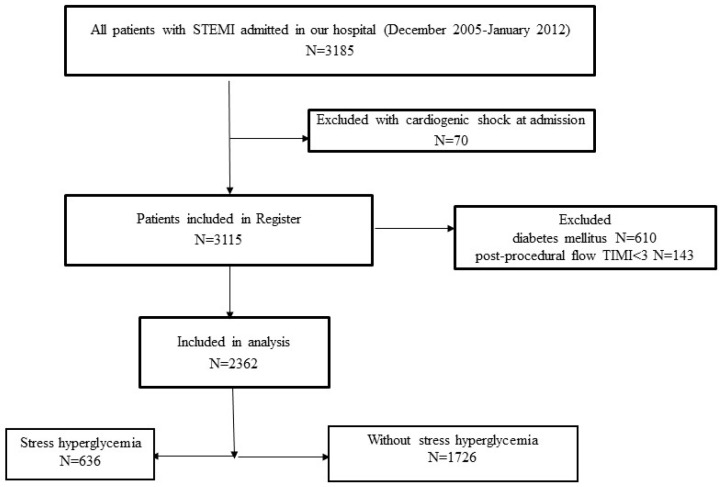
The flowchart of the patient selection.

Patients with cardiogenic shock at admission were excluded from the Register. For the purpose of this study, we excluded (1) patients with DM—patients with previous DM (according to medical history, patients on current antidiabetic therapy), as well as patients with new-onset DM diagnosed during hospitalization—and (2) patients with postprocedural TIMI 0, 1, and 2 flow through the infarct-related artery (IRA). The flowchart of patient selection is presented in Figure 1.

Coronary angiography was performed via the femoral approach. Primary PCI and stenting of the IRA were performed using the standard technique. Loading doses of aspirin (300 mg) and clopidogrel (600 mg) were administered to all patients before pPCI. Selected patients were also given the GP IIb/IIIa receptor inhibitor during the procedure. After pPCI, patients were treated according to the current guidelines.

Demographic, baseline clinical, laboratory, angiographic, and procedural data were collected and analyzed. Blood samples for laboratory analyses were taken at hospital admission before pPCI. SH at hospital admission (and before pPCI) was defined as a plasma glucose level higher than 7.8 mmol/L [20]. An echocardiographic examination was performed in the first three days after the intervention (pPCI). The left ventricular ejection fraction (EF) was assessed according to the biplane method. We classified EF as preserved (EF ≥ 50%), moderately reduced (EF 40–49%), and reduced (EF < 40%). Baseline kidney function (at admission) was assessed using the Modification of Diet in Renal Disease equation and a value of estimated glomerular filtration rate (eGFR) below 60 mL/min/m^2^ was considered as reduced kidney function.

Patients were followed up at eight years after their enrolment in the Register. Follow-up data were obtained through telephone interviews and during outpatient visits. We analyzed all-cause mortality and the composite endpoint—major adverse cardiovascular events (MACE), which included cardiovascular death, nonfatal reinfarction, nonfatal ischemic stroke, and target vessel revascularization (TVR). The cause of death in patients was obtained from death certificates or discharge forms (if the patient was hospitalized). Cardiovascular death included any death due to a proximate cardiac cause (myocardial infarction, low-output heart failure, fatal arrhythmia, or sudden death) and death caused by noncoronary vascular causes, such as cerebrovascular disease [19]. Nonfatal recurrent myocardial infarction was defined according to the Fourth Universal Definition for Myocardial Infarction [21]. TVR was defined as ischemia-driven percutaneous revascularization of the target vessel performed for restenosis or other complications. Stroke was defined as a new onset of focal or global neurological deficit lasting more than 24 h. Computed tomography (CT) was used to diagnose (ischemic) stroke. The Emergency Center neurologist was responsible for the diagnosis and treatment of stroke [19].

### 2.2. Ethics

The study protocol was approved by the Ethics Committee of the University of Belgrade Faculty of Medicine (approval number 470/II-4, 21 February 2008). The study was conducted in keeping with the principles outlined in the Helsinki Declaration. Written informed consent was obtained from all patients for their participation in the Register.

### 2.3. Statistical Analysis

Categorical variables were expressed as frequency and percentages, and continuous variables were expressed as the median (med), with 25th and 75th quartiles (IQR). Analysis for normality of data was performed using the Kolmogorov–Smirnov test. Baseline differences between groups were analyzed using the Mann–Whitney test for continuous variables and the Pearson χ^2^ test for categorical variables. The Kaplan–Meier method was used for constructing the probability curves for eight-year mortality and the incidence of MACE, while the difference between patients with and without SH was tested with the Log-Rank test. The Cox proportional hazard model (backward method, with *p* < 0.10 for entrance into the model) was used to identify univariable and multivariable predictors for the occurrence of all-cause mortality and MACE. Two-tailed *p* values of less than 0.05 were considered statistically significant. We used Version 19 of the SPSS statistical software for statistical analysis (SPSS Inc., Chicago, IL, USA).

## 3. Results

Of the 2362 patients analyzed, 636 (26.9%) patients had SH at admission. The mean age of all analyzed patients was 58 (51, 67) years, with 602 (25.5%) patients being female.

As compared with patients with non-SH, patients with SH were older; they presented more often with heart failure, atrial fibrillation, and complete atrioventricular block; they were more likely to have reduced kidney function at admission (eGFR < 60 mL/min/m^2^), multivessel coronary artery disease on initial angiogram, and preprocedural TIMI grade 0 flow through the IRA. Procedural characteristics did not differ among the analyzed groups. Predischarge EF was significantly lower in patients with SH. Baseline characteristics, laboratory, angiographic, and procedural characteristics, in-hospital mortality, and therapy at discharge in patients with and without premature SH are presented in Table 1.

At eight-year follow-up, all-cause mortality and MACE were registered in a total of 139 (5.9%) patients and 263 (11.1%) patients, respectively. Causes of mortality were predominantly cardiovascular; noncardiovascular causes of death (such as cancer, ileus, pneumonia, and dementia) were registered in a total of 15 patients (10.8% of all deaths).

Patients with SH had higher all-cause mortality and lower composite endpoint MACE. Nonfatal recurrent infarction was related to a new lesion site in 20 (54%) patients with SH at admission and in 29 (42.7%) patients with non-SH, *p* = 0.001.

**Figure 2:**
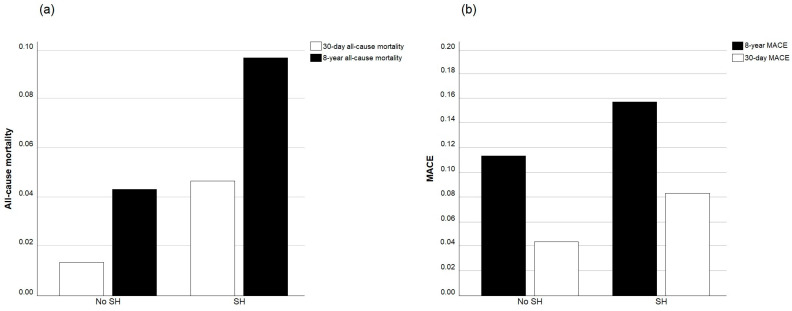
Mortality (**a**) and MACE (**b**) in patients with and without SH.

All-cause mortality and MACE in patients with and without SH are presented in Table 2 and in Figure 2.

**Figure 3:**
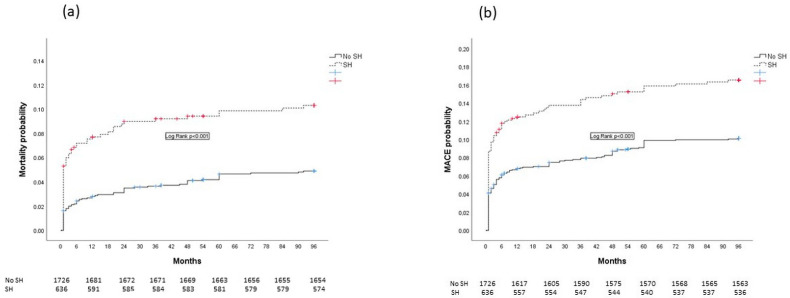
Kaplan–Meier curves showing 8-year all-cause mortality (curve (**a**)) and MACE (curve (**b**)) in analyzed patients.

Kaplan–Meier curves showing eight-year all-cause mortality (curve a) and MACE (curve b) are presented in Figure 3.

After adjustment for confounders, SH at admission was an independent predictor of all-cause mortality and MACE in short-term and long-term follow-up. Predictors for the occurrence of short- and long-term mortality and MACE are presented in Table 3 and Table 4.

## 4. Discussion

Our results showed that more than a quarter of STEMI patients without DM had SH at admission. During eight-year follow-up, mortality and composite endpoint MACE were significantly higher in these patients, as compared to patients without SH, but the incidence of nonfatal recurrent ischemic events was not significantly different between these two groups of patients. The cause of nonfatal recurrent myocardial infarction in patients with SH at admission was predominantly the development of new lesion sites. SH at admission was an independent predictor of short- and long-term mortality and composite end-point MACE, and its negative prognostic impact was stronger in short-term follow-up.

Baseline characteristics and the incidence of SH in our patients are in keeping with data found in the literature, which states that the incidence of SH ranges from 20 to 50%, depending on the analyzed population and the glycemic value by which SH is defined [1,3,6,8,22,23]. In a study by Khalfallah et al., SH at any time during hospitalization (the glycemia level was defined in the same way as in our study) was registered in 16.8% of all analyzed STEMI and nondiabetic patients, while, in the study by Ekmekci et al., wherein STEMI patients treated with pPCI were also included, the incidence of hyperglycemia higher than 118 mg/dL at admission was registered in more than 50% of patients [24]. It should be noted, however, that this study also included patients with DM. In the study by Nakamura et al., hyperglycemia > 11.1 mmol/L was registered in 31% of patients with STEMI and in 15% of nondiabetic patients [25].

### 4.1. Prognostic Impact of SH

Regarding the prognostic influence of SH in patients with AMI, the question can be raised as to whether it is a true independent indicator of adverse events or just a marker of high-risk AMI [8]. In support of the independent prognostic influence of hyperglycemia are the findings of a large number of studies whose results show that SH is an independent predictor of mortality and/or other adverse events independently of the presence of DM in patients with ACS in general but also in patients with AMI with nonobstructive coronary artery disease (MINOCA) [1,2,6,12,14,24,26,27,28]. Data on the negative prognostic impact of SH in STEMI patients are primarily found in studies from the thrombolytic era but are also reported in studies conducted in the era of pPCI. The results of these studies have shown that hyperglycemia (defined by different cut-off values: >6.1 mmol/L or even >11.1 mmol/L) is a strong independent predictor of mortality, i.e., the occurrence of MACEs after STEMI in nondiabetic patients, and the independent and negative prognostic impact of SH is the strongest in short-term follow-up [5]. This finding is explained by the fact that, in nondiabetic patients, SH is a transient disorder of glycoregulation, which gets back to normal after the acute phase of the disease (infarction) passes. Therefore, the negative impact of SH weakens in long-term follow-up. Our results are consistent with these findings but show that the impact of SH at admission persists for up to eight years of follow-up.

In a study by Eitel et al., hyperglycemia was found to be an independent predictor of MACE in patients with STEMI who were treated with pPCI. It was also found that the risk of MACE starts to increase with glycemic values above 7.8 mmol/L in nondiabetic patients (which is in keeping with our findings), while, in patients with diabetes, the risk of the occurrence of MACE starts to increase when the glycemic value is higher than 11.1 mmol/L [7].

In the study by Khalfallah et al., which included patients with STEMI treated with pPCI, it was found that SH in nondiabetic patients was an independent predictor of three-month mortality. Also, in this study, there was no difference in the incidence of nonfatal reinfarction and stroke, which is identical to our findings [1]. Unlike our study, this study also included patients with the slow-flow/no-reflow phenomenon, but this did not affect the findings—in addition to postprocedural TIMI 0–2 flow, SH was also an independent predictor of mortality in three-month follow-up [1].

In a study by Ekmekci et al., the prognostic impact of hyperglycemia at admission on in-hospital occurrence of MACE (cardiovascular mortality, reinfarction, and repeat TVR) was analyzed in patients with STEMI treated with pPCI. In this study, the highest in-hospital mortality was registered in the group of patients with a glycemia level higher than 145 mg/dL. The incidence of reinfarction and ITVR was significantly higher in the group with a glycemia level higher than 145 mg/dL, as compared to the group with a glycemia level lower than 118 mg/dL [24]. The difference in the incidence of MACE is predominantly due to higher mortality, while there was no difference in the incidence of reinfarction and target vessel revascularization. Also, in the cited study, it was found, in multivariate logistic regression, that a glycemia level higher than 145 mg/dL was a strong independent predictor of in-hospital mortality [24].

In a study by Wei et al., it was found that a higher fasting blood glucose level had an adverse prognostic impact on the occurrence of composite MACE during hospitalization and during one-year follow-up of patients with STEMI treated by pPCI and patients with NSTEMI who had undergone early PCI [2]. In contrast to our study, in this study, both patients with DM and nondiabetic patients were analyzed and fasting blood glucose levels were analyzed, while, in our study, the glycemia level was analyzed only at admission, before myocardial revascularization.

In the study by Stalikas et al., wherein 309 patients with STEMI were included, it was found that the glycemic value at admission was independently associated with an increased risk of the occurrence of adverse cardiovascular and cerebrovascular events during an average follow-up period of 1.7 years [29].

It should be noted that there is a somewhat smaller number of studies whose results show that the negative prognostic impact of SH (defined by different cut-off values) after AMI exists only in patients with DM and not in patients without DM. In a study by Ferreira et al., it was found that, in patients with STEMI, hyperglycemia > 213 mg/dL at admission had a negative prognostic impact on 12-year mortality only in patients with diabetes, while the prognosis of patients with DM and a glycemia level of <213 mg/dL at admission as well as the prognosis of nondiabetic patients with a glycemia level of >143 mg/dL at admission and of nondiabetic patients without hyperglycemia at admission did not differ [8]. A study by Kumar et al. had a similar finding establishing that acute hyperglycemia in patients without DM is not an independent predictor of in-hospital mortality, rather, that it is the case only with hyperglycemia in patients with DM [6].

### 4.2. Possible Mechanisms of Adverse Impact of SH in Patients with AMI

The prognostic impact of SH in patients with STEMI is complex and may be direct or indirect. One of the possible indirect effects is that SH is an independent predictor of the slow-flow/no-reflow phenomenon through the IRA [1,17,30]. This is why we decided to include only patients with postprocedural TIMI 3 flow in our analysis, which eliminated the strong negative prognostic impact of poor TIMI flow in our patients.

Other possible direct mechanisms of the prognostic impact of SH may be the consequence of the acute release of catecholamines, cytokines, cortisol, etc., thus leading to oxidative stress, endothelial dysfunction, inflammation, apoptosis, and the state of hypercoagulability, which can affect the size of the infarction despite a good TIMI flow through the IRA [1,2,3,4,6,7,12,14,15,17].

The proinflammatory state in the body in nondiabetic patients further destabilizes insulin secretion and reinforces insulin resistance, which, in turn, further increases blood glucose levels [30]. Some authors have stated that an acute increase in the blood glucose level is more harmful than a chronically elevated glucose level [17], i.e., that the inflammatory response of the body is much more pronounced in nondiabetic patients than in diabetic patients. Interestingly, it was also found that if glucose level remained elevated for a longer period of time, plasma cytokine concentrations gradually returned to normal [15]. These findings suggest that acute hyperglycemia but not sustained elevation of blood glucose level exaggerates inflammation [31]. In our study, we also found that patients with SH had higher C-reactive protein (CRP) level than patients without SH. The negative prognostic impact of SH is believed to be ‘masked’ in patients with DM because the presence of DM itself negatively affects the prognosis after AMI [15,16]. In support of these findings are the results of a large substudy of the HORIZONS-AMI trial, wherein 3405 patients with STEMI were treated with pPCI and where acute hyperglycemia was found to be strongly associated with mortality in nondiabetic patients, and this association was found to be stronger, i.e., more pronounced as compared to the association between hyperglycemia and mortality in patients with DM [32].

### 4.3. Clinical Implications

The results of our study can add to existing knowledge regarding the prognostic impact of SH in nondiabetic patients with STEMI, who were treated with primary PCI. Our findings underscore the importance of determining the blood glucose level in every patient with STEMI and even in those in whom successful PCI was performed. Given that glucose levels are tested routinely and that this is a cheap and readily available testing method, knowing the negative impact of SH can, together with other well-known predictors of poor prognosis after STEMI, provide additional help in risk stratification in these patients.

### 4.4. Study Limitations

Several limitations to our study should be mentioned. This study is unicentric and observational, but it is controlled, prospective, and has included consecutive patients, limiting possible selection bias. Patients included in the study were hospitalized between 2005 and 2012. Patients with cardiogenic shock at admission were excluded from our Register. All patients were treated with clopidogrel. There were no patients treated with more recently developed antiplatelet drugs (ticagrelor and prasugrel were not available for routine administration to patients at the time of their entry into the Register). This may have influenced the patients’ prognosis, i.e., reduced the occurrence of cardiovascular mortality or the incidence of nonfatal ischemic events. Coronary angiography and concomitant PCI were performed via the femoral approach. The radial approach was not used in routine clinical practice at the time when the patients were enrolled into the Register. We analyzed only the glycemia level at admission and we were unable to consider the content and time of the last meal before collecting the blood sample. We did not analyze the SH ratio because we did not determine HgA1c in every patient without DM. However, HgA1c is not a routine laboratory assessment for STEMI patients (especially without DM) in most clinical setups, while testing the blood glucose level is easy to perform and is a routine laboratory test carried out before pPCI in emergency departments [6]. On the other hand, data can be found in the literature stating that the area under the curve for the stress hyperglycemia ratio (SHR) is somewhat lower for predicting intrahospital death in patients with STEMI (AUC = 0.675; (95%CI 0.598–0.752)) [30], while the area under the curve (AUC) for random glucose level is 0.789 (0.759–0.816) for predicting in-hospital death [26]. This study was not designed to evaluate whether changing pharmacological treatment during follow-up would have an impact on long-term outcomes in the analyzed patients.

## 5. Conclusions

A significant number of STEMI patients without DM had SH at admission. Nondiabetic patients with SH at admission had higher eight-year all-cause mortality and composite end-point MACE. The incidence of nonfatal adverse ischemic events did not differ between patients with SH and those without SH. Despite successful revascularization, SH at admission was an independent predictor of short-term and long-term (up to eight years) all-cause mortality and MACE, along with other well-known and significant predictors such as older age, Killip class > 1, EF, and reduced eGFR. This finding emphasizes the importance of close glucose level monitoring in all patients with STEMI regardless of their diabetic status in order to better identify high-risk patients.

## Figures and Tables

**Table 1:** Baseline clinical, laboratory, angiographic, procedural characteristics, therapy at discharge, and intrahospital mortality of the study patients.

Characteristics	SH *N =* 636	No SH *N* = 1726	*p* Value
Age, years med (IQR)	62 (53, 72),	58 (50, 66)	<0.001
Female, *n* (%)	195 (30.6)	402 (23.3)	<0.001
BMI, med (IQR)	26.3 (24.1, 29.2)	26.1 (24.4, 28.9)	0.175
Previous MI, *n* (%)	51 (8.1)	159 (9.2)	0.366
Previous angina, *n* (%)	48 (7.6)	106 (6.1)	0.226
Previous stroke, *n* (%)	22 (3.5)	48 (2.9)	0.729
Hypertension, *n* (%)	422 (66.4)	1070 (62)	0.122
HLP, *n* (%)	368 (57.9)	1054 (61.1)	0.125
Smoking, *n* (%)	333 (52.4)	1051 (60.9)	<0.001
Family hystory, *n* (%)	209 (32.9)	620 (35.9)	0.167
Pain duration, hours med (IQR)	2.5 (1.5, 4.5)	2.5 (1.5, 4)	0.876
Atrial fibrillation on initial ECG, *n* (%)	60 (9.5)	81 (4.7)	<0.001
Complete AV block, *n* (%)	39 (6.2)	60 (3.5)	0.004
Killip class > 1, *n* (%)	97 (15.3)	124 (7.2)	<0.001
Systolic BP at admission, med (IQR)	130 (120, 150)	135 (120, 150)	0.984
Heart rate at admission med (IQR)	80 (70, 90)	76 (70, 88)	0.047
Multivessel disease, *n* (%)	372 (58.5)	885 (51.3)	0.002
3-vessel disease, *n* (%)	171 (26.9)	375 (21.7)	0.008
LM stenosis, *n* (%)	34 (5.4)	98 (5.7)	0.755
Preprocedural flow TIMI 0, *n* (%)	479 (75.3)	1152 (66.7)	<0.001
Stent implanted, *n* (%)	611 (96.1)	1653 (95.8)	0.747
Acute stent thrombosis, *n* (%)	11 (1.8)	11 (0.7)	0.15
Glicoprotein IIb/IIIa inhibitor, *n* (%)	247 (38.8)	634 (36.7)	0.358
CK MB, med (IQR)	2472 (1321, 4608)	1804 (921, 3376)	<0.001
Troponin I, med (IQR)	75 (30, 116)	50.3 (20.1, 83.6)	<0.001
WBC count, med (IQR)	12 (10.1, 14.1)	11.4 (9.1, 14.1)	0.645
Fibrinogen at admission, med (IQR)	3.9 (3.3, 4.9)	4 (3.3, 4.8)	0.360
CRP at admission, med (IQR)	11.7 (3.2, 45.7)	7.8 (2.8, 26.8)	0.036
Hemoglobin at admission g/L, med (IQR)	141 (132, 151)	143 (133, 153)	0.245
eGFR < 60 mL/min/m^2^, *n* (%)	116 (18.3)	201 (11.6)	<0.001
EF, med (IQR)	45 (40, 52)	50 (42, 55)	<0.001
EF < 40%, *n* (%)	105 (16.1)	165 (9.6)	<0.001
EF 40–49%, *n* (%)	202 (31.8)	493 (28.6)	<0.001
EF ≥ 50%, *n* (%)	306 (48.1)	1020 (59.1)	<0.001
Therapy at discharge *			
Beta blockers, *n* (%)	523 (82.2)	1490 (86.4)	0.575
ACE inhibitors, *n* (%)	480 (75.5)	1390 (80.5)	0.554
Statin, *n* (%)	563 (88.5)	1279 (74.1)	0.176
Diuretic, *n* (%)	112 (17.6)	217 (12.6)	<0.001
Calcium antagonist, *n* (%)	23 (3.6)	57 (3.3)	0.101
Amiodarone, *n* (%)	55 (8.7)	12 (0.7)	<0.001
In-hospital mortality, *n* (%)	21 (3.3)	29 (1.7)	<0.001

* All patients were on aspirin and clopidogrel. SH = stress hyperglycemia; med = median; IQR = interquartile range; BMI = body mass index; MI = myocardial infarction; EF = left ventricular ejection fraction; HLP = hyperlipidemia; AV = atrioventricular; HF = heart failure; BP = arterial blood pressure; BBB = bundle branch block on ECG at admission; LM = left main artery; LAD = left anterior descendent; CK-MB = creatine kinase MB isoform; WBC = white blood cell count at admission; CRP = C-reactive protein; eGFR = estimated glomerular filtration rate.

**Table 2:** Mortality and composite end-point MACE in the patients enrolled in this study.

Event	SH *N* = 636	NoSH *N* = 1726	*p* Value
30 days			
All-cause mortality, *n* (%)	3 (0.4)	4 (0.2)	<0.001
MACE, *n* (%)	15 (2.5)	25 (1.5)	<0.001
Cardiovascular death, *n* (%)	3 (0.4)	4 (0.2)	<0.001
Nonfatal recurrent infarction, *n* (%)	5 (0.8)	10 (0.6)	0.281
TVR, *n* (%)	13 (2)	28 (1.6)	0.578
Nonfatal stroke, *n* (%)	2 (0.3)	3 (0.2)	0.281
8 years			
All-cause death, *n* (%)	62 (9.7)	77 (4.2)	<0.001
MACE, *n* (%)	100 (15.7)	163 (9.4)	<0.001
Cardiovascular death, *n* (%)	58 (9)	66 (3.8)	<0.001
Nonfatal recurrent infarction, *n* (%)	37 (5.8)	68 (4)	0.051
TVR, *n* (%)	44 (6.9)	102 (5.9)	0.465
Nonfatal stroke, *n* (%)	7 (1.1)	21 (1.2)	0.835

SH = stress hyperglycemia; MACE = major adverse cardiovascular events; TVR = target vessel revascularization.

**Table 3:** Predictors for 30-day all-cause mortality and MACE (Cox regression model) in all analyzed patients.

	Univariable Analysis		Multivariable Analysis	
	HR (95%CI)	*p* Value	HR (95%CI)	*p* Value
**All-Cause Mortality**				
Age, years	1.01 (1.00–1.02)	<0.001	1.01 (1.00–1.02)	<0.001
EF%	0.98 (0.96–0.99)	<0.001	0.95 (0.92–0.99)	0.010
SH	3.14 (1.28–7.65)	0.012	2.19 (1.16–4.18)	0.016
Killip class > 1 at admission	2.16 (1.41–3.18)	<0.001	2.13 (1.38–3.29)	<0.001
3-vessel disease	1.92 (1.06–3.83)	0.042		
		MACE		
Age, years	1.02 (1.03–1.05)	<0.001	1.01 (1.0–1.03)	0.001
EF%	0.96 (0.93–0.98)	<0.001	0.94 (0.92–0.96)	0.002
Killip class > 1	2.71 (1.52–4.90)	0.001	2.66 (1.48–4.74)	0.001
SH	2.42 (1.16–6.09)	0.030	1.99 (1.03–3.85)	0.048
Previous MI	1.96 (1.16–6.09)	0.040		

EF = left ventricular ejection fraction; SH = stress hyperglycemia; CKD = chronic kidney disease; MI = myocardial infarction.

**Table 4:** Predictors for 8-year all-cause mortality and MACE (Cox regression model) in all analyzed patients.

	Univariable Analysis		Multivariable Analysis	
	HR (95%CI)	*p* Value	HR (95%CI)	*p* Value
**All-Cause Mortality**				
Age, years	1.04 (1.02–1.06)	<0.001	1.04 (1.01–1.07)	0.012
EF%	0.87 (0.85–0.91)	<0.001	0.87 (0.85–0.90)	<0.001
Killip class > 1 at admission	4.26 (2.29–7.96)	<0.001	3.89 (2.04–7.42)	<0.001
eGFR < 60 mL/min/m^2^	1.98 (1.02–3.86)	0.045	1.89 (1.16–3.67)	0.054
SH	1.54 (1.05–2.80)	0.045	1.49 (1.10–2.03)	0.048
		MACE		
Age, years	1.02 (1.0–1.03)	<0.001	1.02 (1.01–1.03)	<0.001
EF%	0.94 (0.93–0.96)	<0.001	0.94 (0.93–0.95)	<0.001
Killip class > 1 at admission	1.48 (1.15–2.16)	0.025	1.45 (1.03–2.01)	0.036
SH	1.39 (1.08–1.91)	0.042	1.35 (1.01–1.89)	0.048

EF = left ventricular ejection fraction; eGFR = estimated glomerular filtration rate; MI = myocardial infarction; SH = stress hyperglycemia.

## Data Availability

The data presented in this study are available on request from the corresponding author.
